# The effect of single-nucleotide polymorphisms within heat shock protein beta 1 on beef quantity in Korean native steers

**DOI:** 10.5194/aab-63-417-2020

**Published:** 2020-11-17

**Authors:** Jung-Keun Suh, Jae-Sung Lee, Hongsik Kong, Yoonseok Lee, Hong-Gu Lee

**Affiliations:** 1 Department of Animal Science and Technology, Sanghuh College of Life Sciences, Konkuk University, Seoul, 05029, Republic of Korea; 2 Team of An Educational Program for Specialists in Global Animal Science, Brain Korea 21 Plus Project, Sanghuh College of Life Sciences, Konkuk University, Seoul, 05029, Republic of Korea; 3 Department of Biotechnology, Hankyung National University, Anseong-si, Gyeonggi-do, 17579, Republic of Korea; 4 Center for Genetic Information, Hankyung National University, Anseong-si, Gyeonggi-do, 17579, Republic of Korea

## Abstract

Heat shock protein beta 1 (*HSPB1*), a member of the heat-shock family of protein, is a relatively small (27 kDa) molecular chaperone protein associated with cellular development. The relationship between *HSPB1* expression and muscle growth in beef cattle has previously been reported, but there have been no reports of DNA markers related to meat quantity in Korean native steers. Therefore, the aim of this study was to evaluate the relationship of single-nucleotide polymorphisms (SNPs) within *HSPB1* in terms of the carcass traits related to muscle growth in Korean native steers. Through direct sequencing, we discovered three SNPs: g.111 T > C SNP (rs208395876) and g.2548 C > G SNP (rs483014585) were respectively located in 5${}^{\prime }$ UTR (untranslated region) and 3${}^{\prime }$ UTR. Further, g.2352 T > C SNP (rs110832311) was located in the adjacent region of the RNA splicing site. The least square means of steers with a CC genotype of g.111 T > C SNP had a significantly higher meat ratio (P = 0.04), while the least square means of steers with a CC genotype of g.2352 T > C SNP had a significantly higher meat ratio (P = 0.002) and lower back-fat thickness (P = 0.004) than those of the other genotype. Moreover, although the least square means of steers with CC-CC, CT-CC, and TT-CC genotypes were significantly decreased for back-fat thickness, they were significantly increased for the meat ratio. Therefore, our results suggested that g.111 T > C SNP and g.2352 T > C SNP could be a causal mutation related to an adipose metabolism in Korean cattle steer.

## 1 Introduction

Recently, several studies have evaluated the relationship between heat-shock protein beta 1 (*HSPB1*) and muscle growth (Dubińska-Magiera et al., 2014; Hamelin et al., 2006). *HSPB1* is a 27 kDa small heat-shock protein that is expressed in many vertebrate tissues, particularly muscle. The general functions of *HSPB1* in muscle are to protect cells from physiological stress, inhibit cell death, and chaperone activity (Arrigo, 2017). In previous studies, *HSPB1* has also been shown to be relevant in terms of feed efficiency (Jung et al., 2017). *HSPB1* is also involved in muscle development in many species, including those reported in Dubińska-Magiera et al. (2014). *HSPB1* has been shown to be related to muscle hypertrophy in vivo (Hamelin et al., 2006). In a recent study, the expression of *HSPB1* was found to be significantly increased during bovine myogenic differentiation compared to in the un-differentiation stage in myogenic cells, and it was also shown to be regulated by androgen-mediated myogenesis (Zhang et al., 2012, 2014).

**Figure 1:**
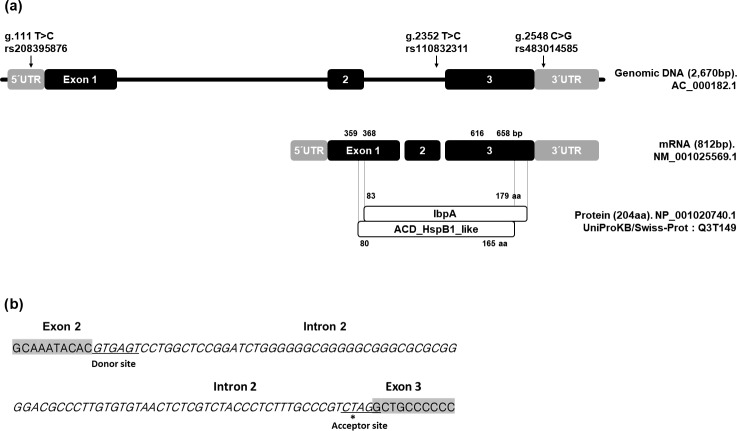
Position of SNPs within the *HSPB1* gene. **(a)** Black and grey boxes represent UTR and exon, respectively. **(b)** Position of g.2352 SNP (${}^{*}$) in RNA splicing site. Grey boxes represent exon 2 and exon 3 of *HSPB1* gene. The italic letters represent the intron 2 region. Underscores refer to the donor and acceptor sites of the RNA splicing site.

In the beef industry, efficient muscle growth and development in cattle is critical for meat production (He et al., 2017).

Myogenesis is regulated by several myogenic genes, such as *MyoD*, *Myogenin*, *MRF4*, and *Desmin* (Charge and Rudnicki, 2004). These myogenic marker genes are known to be upregulated during myogenic differentiation (Sweetman, 2012). *HSPB1*, a major factor of actin polymerization in muscle, is known to play a role in maintaining muscle structure (Sugiyama et al., 2000). *HSPB1* also shows chaperone function, including preventing protein degradation, inhibiting muscle atrophy, and stabilizing muscle protein (Tucker and Shelden, 2009). It has been speculated that *HSPB1* enhances muscle development by protecting muscle proteins (Perng et al., 1999). In a recent study, the inhibition of *HSPB1* expression during myogenesis was shown to repress the expression of *MyoD*, *Myogenin*, and *Desmin*; formation of myotubes; and protein synthesis (Kim et al., 2018). A relationship has also been reported between *HSPB1*-10 expression and muscle growth in beef cattle, but there have been no reports of DNA markers related to meat quantity in Korean native steers. Therefore, the aim of this study was to evaluate the relationship of SNPs within *HSPB1* with carcass traits related to muscle growth in Korean native steers.

## 2 Materials and methods

### 2.1 Animals, DNA extraction, SNP discovery

A total of 192 Korean cattle (body weight BW = 464.68 ± 45.09 kg; 32.18 months old [SD1.0]) raised in Pyeongchang (Gangwon, Republic of Korea) were used in this study. All of the steers were maintained under constant environmental conditions, such as having access to two types of commercial feeds at six feedlots. Muscle tissues were sampled from slaughtered individuals. Therefore, there is no certificate of animal ethics. Genomic DNA was extracted from longissimus dorsi muscle tissue using a LaboPass™ tissue mini kit (Cosmo Genetech, Seoul, Korea). In order to discover SNPs, the bovine *HSPB1* sequence was obtained from the NCBI database (AC_000182.1). The primer sequence was designed using NCBI Premer-BLAST based on the selected polymorphism sites, and the primer information is shown in Table 1. The sequencing was performed as outlined in a previous study (Lee et al., 2010), and SNPs were discovered using the Sequencer v5.2.4 program (Gene Codes Corp., Ann Arbor, MI). In order to map the functional SNPs on DNA, mRNA, and protein sequences, they were aligned using the Graphical View Legend on the NCBI database.

**Table 1:** The single effect of g.111 T > C SNP and g.2352 T > C SNP within *HSPB1* gene on carcass traits in a commercial Korean native steer population.

Carcass traits	Genotype (number of animals)							
	g.111 T > C SNP				g.2352 T > C SNP			
	CC (6)	CT (66)	TT (118)	p value	CC (42)	CT (91)	TT (57)	p value
Back-fat thickness, mm	12.5 ± 1.65	13.15 ± 0.51	14.45 ± 0.38	0.084	12.09 ± 0.63${}^{a}$	14.48 ± 0.42${}^{b}$	14.31 ± 0.53${}^{b}$	0.004
Rib-eye area, cm${}^{2}$	98 ± 3.64	94.92 ± 1.11	94.23 ± 0.84	0.562	96.31 ± 1.4	93.96 ± 0.95	94.41 ± 1.18	0.362
Carcass weight, kg	465.18 ± 17.2	456.29 ± 5.27	464.69 ± 3.99	0.428	459.64 ± 6.65	464.92 ± 4.5	461.64 ± 5.63	0.776
Meat ratio${}^{*}$	65.17 ± 1.23	64.58 ± 0.38	63.50 ± 0.29	0.04	65.37 ± 0.47${}^{a}$	63.44 ± 0.32${}^{b}$	63.68 ± 0.4${}^{b}$	0.002

${}^{a,\;b}$ Means with the same superscript in the same row for each quality are not significantly different (P < 0.05). ${}^{*}$ Meat ratio = 68.184–[0.625 × back-fat thickness (mm)] + [0.13 × rib-eye area (${\mathrm{cm}}^{2})$]–[0.024 carcass weight (kg)]. The Korean native cattle meat ratio has an additional 3.23 added after calculation.

**Table 2:** The multiple effect of genotype combination of pairwise SNPs on carcass traits in commercial Korean native steer population.

Carcass trait	Genotype (number of animals)						p value
	CC-CC (6)	CT-CC (21)	CT-CT (45)	TT-CC (15)	TT-CT (46)	TT-TT (57)	
Back-fat thickness, mm	12.49 ± 1.61${}^{a}$	12.22 ± 0.87${}^{a}$	13.54 ± 0.59${}^{\mathrm{ab}}$	11.73 ± 1.04${}^{a}$	15.39 ± 0.59${}^{b}$	14.32 ± 0.53${}^{\mathrm{ab}}$	0.007
Rib-eye area, cm${}^{2}$	98 ± 3.65	96.82 ± 1.96	94.04 ± 1.35	94.84 ± 2.36	93.85 ± 1.33	94.4 ± 1.19	0.745
Carcass weight, kg	465.18 ± 17.2	447.49 ± 9.25	461.55 ± 6.35	472.07 ± 11.1	467.43 ± 6.28	461.76 ± 5.65	0.521
Meat ratio${}^{*}$	65.17 ± 1.2${}^{a}$	65.62 ± 0.65${}^{a}$	64.12 ± 0.45${}^{\mathrm{ab}}$	65.08 ± 0.78${}^{\mathrm{ab}}$	62.77 ± 0.44${}^{b}$	63.67 ± 0.39${}^{\mathrm{ab}}$	0.004

${}^{a,\;b}$ Means with the same superscript in the same row for each quality are not significantly different (P < 0.05). ${}^{*}$ Meat ratio = 68.184–[0.625 × back-fat thickness (mm)] + [0.13 × rib-eye area (${\mathrm{cm}}^{2})$]–[0.024 carcass weight (kg)]. The Korean native cattle meat ratio has an additional 3.23 added after calculation.

### 2.2 SNP genotyping and statistical analysis

Large-scale SNP genotyping was performed commercially using the Fluidigm^®^ SNP™ Type assay as described in a previous study (Oh et al., 2018). In order to evaluate the association SNPs and carcass traits, the data were analyzed using the general linear model (GLM) of SPSS v. 22 (IBM, USA). The model is as follows:

1 Yijkl=μ+Pi+GSj+SNPk+βagel+eijkl,

where Yijkl is a phenotype of the carcass trait, μ is the overall mean for each trait, Pi is the feed type in farms, GSj is the random effect of the sire, SNPk is the fixed effect of SNP genotype, βagelis the covariance of days bred, and eijkl is the random error.

## 3 Results and discussion

Bovine *HSPB1* has been shown to be closely associated with cell growth and bovine myogenesis in certain types of muscles. Thus, based on the results reported by Kim et al. (2018), we found that SNPs, which are directly regulated by gene expression, were discovered, and then we identified the relationship of their SNPs with beef quantity.

The position of SNP in DNA, RNA, and the protein sequence of the *HSPB1* gene as well as their alignments are shown in Fig. 1. In the present study, we discovered three SNPs using direct sequencing. These SNPs were located in 5${}^{\prime }$ UTR, intron 2, and 3${}^{\prime }$ UTR; g.111 T > C SNP (rs208395876) and g.2548 C > G SNP (rs483014585) are in 5${}^{\prime }$ UTR and 3${}^{\prime }$ UTR, while g.2352 T > C SNP (rs110832311) is in the region adjacent to the RNA splicing site. However, SNPs were not discovered in the exon region.

The multiple and single effects of SNPs within *HSPB1* on carcass traits, such as beef quantity, are shown in Tables 1 and 2. A previous study (unpublished data) demonstrated that the g.2548 C > G SNP (rs483014585) had no effects on carcass traits, and we thus removed the related data from the present study, as we could not use it as evidence to support our result. As shown in Table 1, g.111 T > C SNP was significantly associated with meat ratio (P > 0.05). The least square mean in the steer group with a CC genotype of g.111 T > C SNP was significantly higher than that in the steer group with other genotypes. Moreover, g.2352 T > C SNP was significantly associated with back-fat thickness and meat ratio (P < 0.01). Regarding back-fat thickness, the least square mean in the steer group with the CC genotype of g.2352 T > C SNP was significantly lower than that in the steer group with other genotypes. On the other hand, in terms of meat ratio, this group was significantly higher than that in the steer group with other genotypes.

As shown in Table 2, consistent with the results of a single effect, the combination genotype of g.111 T > C and g.2352 T > C SNPs was significantly associated with back-fat thickness and meat ratio (P < 0.01). The steer group with combination genotypes of CC-CC, CT-CC, and TT-CC had a low least square mean for back-fat thickness compared to the steer group with other combinations. On the other hand, the steer group with the combination genotype had the highest least square mean for the meat ratio.

*HSPB1* is expressed in many vertebrate tissues, particularly muscle. In a previous study, Zhang et al. (2012) demonstrated that the *HSPB1* expression level was higher in the skeletal muscle of bulls than that of steers. Thus, in this study, the steer group in Korean cattle was used. As shown in Tables 1 and 2, although there were no significant differences between these SNPs and the rib-eye area, the mean in the group with the CC genotype of g.2352 T > C SNP and combination genotypes of CC-CC, CT-CC, and TT-CC were found to be numerically higher than that in other groups.

The cellular development associated with adipose tissue growth involves both cellular hypertrophy (increase in size) and hyperplasia (increase in number). Rajesh et al. (2010) reported that *HSPB1* interact with insulin-like growth factor receptor 1 and its signal transducer, the serine/threonine kinase Akt, which together modulate an adipocyte metabolism. Specifically, the previous results suggested that *HSPB1* was negatively correlated with an adipose metabolism (Kim et al., 2011). Therefore, our results regarding the least square mean of back-fat thickness and the rib-eye area were found to be similar.

In a previous study, it was reported that g.111. T > C SNP (rs208395876) was functional. In particular, our results identified that g.2352 T > C SNP was located in the acceptor site of the RNA splicing region.

Therefore, our results suggested that g.111 T > C SNP and g.2352 T > C SNP could be causal mutations related to an adipose metabolism in Korean cattle steer.

## 4 Conclusions

We discovered three SNPs, including g.111 T > C, g.2352, and g.2548, which are respectively located in 5${}^{\prime }$ UTR, intron 2, and 3${}^{\prime }$ UTR of the *HSPB1* protein. Animals with a CC genotype of g.111 T > C SNP had a significantly higher meat ratio, and animals with a CC genotype of g.2352 T > C SNP had a significantly higher meat ratio and lower back-fat thickness than those of other genotypes. Moreover, for the combination of g.111 T > C and g.2352 T > C SNPs, the CC-CC, CT-CC, and TT-CC genotypes' back-fat thicknesses were found to decrease while the meat ratios increased. In particular, a g.2352 T > C SNP was found to be located in the acceptor site of the RNA splicing site. Therefore, our results indicate that it could be a causal mutation related to an adipose metabolism in Korean cattle steer.

## Data Availability

The original data of the paper are available from the corresponding author upon request.

## Appendix A

**Table A1:** Primer information used in this study.

SNP	dbSNP no.	Primer sequence		Tm	Product	Primer sequence for Fluidigm SNP genotyping${}^{*}$	
				(${}^{\circ }$)	size (bp)		
g.111 T > C	rs208395876	F	AAGGTTCCAGATGTGGGCAG	62	781	ASP1	CGCCCGCCACTTCTCC
		R	ACCAGGGGTTGGGCGAAGAG			ASP2	CGCCCGCCACTTCTCT
						LSP	GGCCATGCTGGCTGGTC
						STA	CCATAAAAGCGCTGCGGG
g.2352 T > C	rs110832311	F	CAGTCTCGGGCACCCAGATC	60	783	ASP1	CTACCCTCTTTGCCCGTCT
		R	AGTGACGGATGGCACTGCAC			ASP2	ACCCTCTTTGCCCGTCC
						LSP	GGGTGGGGTCCACACCG
						STA	GACGCCCTTGTGTGTAACT
g.2548 C > G	rs483014585	F	CAGTCTCGGGCACCCAGATC	60	783	ASP1	AACAGCCGGAAAACAAGTAAAGAC
		R	AGTGACGGATGGCACTGCAC			ASP2	GAACAGCCGGAAAACAAGTAAAGAG
						LSP	TGGGCCGCTGGGCTAA
						STA	CCCCGAAGCCGGGAAG

${}^{*}$ ASP1: allele specific primer 1, ASP2: allele specific primer 2, LSP: locus specific primer, STA: specific target amplification.
